# Supplementary material for: Coronavirus Disease 2019 (COVID-19): A Modeling Study of Factors Driving Variation in Case Fatality Rate by Country
Source: Int J Environ Res Public Health. 2020 Nov 5;17(21):8189. doi: 10.3390/ijerph17218189 (PMC7664233; doi:10.3390/ijerph17218189)
Supplement: Supplementary file 1 [file ijerph-17-08189-s001.pdf]

Table S1 24 country-level risk factors with potential influence on COVID-19 case fatality rates.

Variable	Variable Description	Source
Population	Country population as of 2020	(1)
Population density	Population density as of 2020	(2)
Percent population over 70	Percent of population over 70 years of age	[1]
Total Cases[a]	Total confirmed COVID-19 cases as of 2020	(1)
Total deaths[a]	Total confirmed COVID-19 deaths as of 2020	(1)
CFR[a]	Case fatality ratio calculated from total cases and total deaths	(1)
Gross Domestic Product	Country GDP as of 2017 in US dollars	(3)
GDP per capita	Country GDP per person as of 2017 in US dollars	(3)
Healthcare expenditure per capita	Healthcare expenditure per person as of 2017 in US dollars	(2)
Scientific production	Scientific Production as of 2016 in US dollars	(2)
Hospital beds per 1000	Hospital beds per 1000 people	(2)
Physicians per 1000	Physicians per 1000 people	(2)
General mortality rate per 100000	Country's general mortality rate per 100000	(2)
Life expectancy	_(mean/median) life expectancy as of 2020	(2)
CT scanners per 1 million	CT scanners per 1 million people as of 2018	(4)
Radiology Specialists	Number of radiologists per country as of 2008	(5)
Radiologists per 1 million	Radiologists per 1 million people calculated from population and radiology specialists	(1, 5)
Total tests[b]	Total tests performed for COVID in a given country	(1)
Tests per thousand[b]	Number of COVID tests in a given country per 1000 people	(1)
Median Age	Median Age of country	(6)
Date of 100th case	Date of 100th confirmed COVID-19 case	(1)
Date of first government[c]	Date of first government intervention on its citizenry	[d]
Days from 100th case to intervention	Number of days from date of 100th case to date of first intervention.	(1)
Air travel activity	Air travel in 1000s of passengers carried over 2018.	(7)

Education	Percent of a population aged 25 to 64 with at least a high-school level degree or equivalent.	(4)
Literacy Rate	Percent of a population which is literate.	(8)
Obesity Prevalence	Percent of a population with a BMI >30 in 2017.	(9)
Prevalence Daily smoking	Prevalence of daily smoked tobacco use in a population in 2012.	(9)
Prevalence tobacco use	Prevalence of any tobacco use in a population in 2017.	(9)
HIV prevalence	Prevalence of HIV in a population in 2017	(9)
COPD	Prevalence of COPD in a population in 2017	(9)
Air pollution	Mean annual exposure (micrograms per cubic meter) in 2017.	(9)

[a] Current as of April 22, 2020.

[b] Current as of April 21, 2020.

[c] Government responses have varied across countries. For purposes of this study, we defined this intervention as the first date when recommendations or legislation was passed limiting gathering size, closing non-essential business, or encouraging social distancing. School closures and international travel bans were not considered.

[d] Dates taken from various news sources and compiled in supplemental data (**Table S2**).

Table S2 Date of first government intervention per aggregation of secondary news sources as indicated.[a]

Country	Date	Source
Belgium	3/18/2020	Belgium Federal Authorities Coronavirus Info(10)
Brazil	3/21/2020	Reuters(11)
France	3/16/2020	The Independent(12)
Germany	3/16/2020	Bayerischer Rundfunk (Bavarian Broadcasting)(13)
Iran	3/24/2020	Reuters(14)
Italy	3/9/2020	Wall Street Journal(15)
Netherlands	3/15/2020	NOS (Dutch Broadcast Foundation)(16)
Spain	3/28/2020	The Guardian(17)
United Kingdom	3/20/2020	NBC(18)
United States	3/21/2020	Wall Street Journal(19)
Australia	3/23/2020	Sydney Morning Herald(20)
Austria	3/16/2020	ORF(21)
Canada	3/23/2020	CTV news(22)
Chile	3/22/2020	National Post(23)
China	1/21/2020	South China Morning Post(24)
Czech Republic	3/14/2020	Government of the Czech Republic(25)
Denmark	3/13/2020	TV2(26)
Ecuador	3/16/2020	Oxford Government Response Tracker - Ecuador(27)[a]
India	3/17/2020	Live Mint (28)
Indonesia	4/10/2020	Detik News(29)
Ireland	3/27/2020	Government of Ireland (30)
Israel	3/11/2020	Jerusalem Post (31)
Japan	3/26/2020	The Asahi Shimbun (32)
Malaysia	3/18/2020	Malay Mail (33)
Mexico	3/23/2020	UNO TV (34)
Norway	3/12/2020	NRK (35)

Pakistan	3/22/2020	Radio Pakistan(36)
Peru	3/15/2020	Gestión (37)
Philippines	3/16/2020	GMA News Online (38)
Poland	3/24/2020	TVN(39)
Portugal	3/19/2020	The Portugal News(40)
Romania	3/25/2020	Romania Ministry of Health (41)
Russian Federation	3/24/2020	Government of the Russian Federation(42)
Saudi Arabia	3/15/2020	Al Arabiya(43)
South Korea	2/24/2020	New York Times(44)
Sweden	3/10/2020	Public Health Agency of Sweden(45)
Switzerland	3/16/2020	Switzerland Federal Office of Public Health(46)
Turkey	4/11/2020	Garda(47)
United Arab Emirates	3/26/2020	Al Arabiya(48)

[a]Obtained by searching for first date of movement restrictions between dates March 1, 2020 and March 31, 2020.

References:

1. M. Roser, H. Ritchie, E. Ortiz-Ospina, Coronavirus Disease (COVID-19) – Statistics and Research (2020) (April 15, 2020).

2. The World Bank, World Bank Open Data (April 25, 2020).

3. M. Roser, Economic Growth. *Our World Data* (2013) (April 29, 2020).

4. Organisation for Economic, Co-operation and Development, OECD data. *theOECD* (April 25, 2020).

5. Y. Nakajima, K. Yamada, K. Imamura, K. Kobayashi, Radiologist supply and workload: international comparison: Working Group of Japanese College of Radiology. *Radiat. Med.* **26**, 455–465 (2008).

6. , World Population Prospects - Population Division - United Nations (April 29, 2020).

7. L. Martelleti, P. Martelleti, Air Pollution and the Novel Covid-19 Disease: a Putative Disease Risk Factor. *SN Compr Clin Med*, 1–5 (2020).

8. M. Roser, E. Ortiz-Ospina, Literacy. *Our World Data* (2016) (April 29, 2020).

9. , Global Health Data Exchange | GHDx (April 29, 2020).

10. , Coronavirus : Phase 2 maintained, transition to the federal phase and additional measures | Coronavirus COVID-19 (April 30, 2020).

11. , Brazil's Sao Paulo to get two-week coronavirus shutdown, Bolsonaro blasts "hysteria." *Reuters* (2020) (April 29, 2020).

12. , Coronavirus: France imposes 15-day lockdown and mobilises 100,000 police to enforce restrictions | The Independent (April 29, 2020).

13. , Katastrophenfall: Diese Regeln gelten in Bayern. *BR24* (2020) (April 29, 2020).

14. , Half of Iranian government employees to work remotely amid coronavirus - Rouhani. *Reuters* (2020) (April 29, 2020).

15. E. S. and G. Legorano, As Virus Spreads, Italy Locks Down Country. *Wall Str. J.* (2020) (April 29, 2020).

16. , De uitzending van 15 maart: Verregaande maatregelen in strijd tegen corona (April 30, 2020).

17. , Spain orders non-essential workers stay home for two weeks | World news | The Guardian (April 29, 2020).

18. S. Meredith, UK PM Boris Johnson announces nationwide lockdown measures, telling cafes, pubs and restaurants to close. *CNBC* (2020) (April 29, 2020).

19. , A Guide to State Coronavirus Reopenings and Lockdowns - WSJ (April 29, 2020).

20. , Drastic bans on pubs, gyms and cinemas in bid to stop coronavirus (April 29, 2020).

21. O. at/Agenturen red, Ausgangsbeschränkungen: Was nun erlaubt ist und was nicht. *news.ORF.at* (2020) (April 29, 2020).

22. , Feds launch ad campaign urging social distancing, hygiene during COVID-19 crisis. *Coronavirus* (2020) (April 29, 2020).

23. , Chile announces nationwide nightly curfew, coronavirus cases hit 632 | National Post (April 29, 2020).

24. , China's credibility on the line as it tries to dispels fears it will cover up spread of Wuhan virus | South China Morning Post (April 29, 2020).

25. , Vláda rozhodla o uzavření všech obchodů kromě prodejen potravin, lékáren, drogerií, čerpacích stanic a některých dalších (April 29, 2020).

26. , Danmark lukker ned: Her er regeringens nye tiltag - TV 2. *nyheder.tv2.dk* (2020) (April 29, 2020).

27. , OxCGRT (April 29, 2020).

28. , Govt calls for social distancing as confirmed coronavirus cases rise to 124 (April 29, 2020).

29. A. Ikhsanudin, DKI Jakarta Akan Perpanjang Masa PSBB. *detiknews* (April 29, 2020).

30. , Gov.ie - Public health measures in place until 5 May to prevent spreading COVID-19 (April 29, 2020).

31. , Israel limits gatherings to 100 people as coronavirus cases climb to 97 - The Jerusalem Post (April 29, 2020).

32. , Prefectures urging residents to avoid travel to virus-hit Tokyo. *Asahi Shimbun* (April 29, 2020).

33. , PM: Malaysia under movement control order from Wed until March 31, all shops closed except for essential services | Malaysia | Malay Mail (April 29, 2020).

34. , México inicia fase 2 por coronavirus; toma estas medidas (2020) (April 29, 2020).

35. P. Kalajdzic, Alle utdanningsinstitusjoner stenges – flere arrangementer og virksomheter får forbud. *NRK* (2020) (April 29, 2020).

36. , GB Govt decides to observe lockdown for indefinite period (April 29, 2020).

37. R. Gestión, Coronavirus en Perú: Gobierno anuncia cuarentena obligatoria por 15 días por coronavirus. *Gestión* (2020) (April 29, 2020).

38. , Duterte orders Luzon-wide "enhanced community quarantine" (April 29, 2020).

39. , Koronawirus w Polsce. Zakaz wychodzenia z domu bez konkretnego powodu i nowe obostrzenia od 25 marca - TVN24 (April 29, 2020).

40. , State of emergency explained (April 29, 2020).

41. , Buletin informativ 25.03.2020 – Ministerul Sănătății (April 29, 2020).

42. , О решениях по итогам заседания оперативного штаба по предупреждению завоза и распространения новой коронавирусной инфекции на территории Российской Федерации (April 29, 2020).

43. , Saudi Arabia closes commercial centers and restaurants to reduce Corona (April 29, 2020).

44. M. Fisher, C. Sang-Hun, How South Korea Flattened the Curve. *N. Y. Times* (2020) (April 29, 2020).

45. , Flera tecken på samhällsspridning av covid-19 i Sverige — Folkhälsomyndigheten (April 29, 2020).

46. F. O. of P. H. FOPH, New coronavirus: Measures, ordinance and explanations (April 30, 2020).

47. , Turkey: Authorities to implement 48-hour lockdown in 31 cities as of April 11 /update 14. *GardaWorld* (April 29, 2020).

48. , Coronavirus: UAE to shut public transport, restrict movement from March 26-29 | Al Arabiya English (April 29, 2020).
